# The structure of a family 110 glycoside hydrolase provides insight into the hydrolysis of α-1,3-galactosidic linkages in λ-carrageenan and blood group antigens

**DOI:** 10.1074/jbc.RA120.015776

**Published:** 2021-01-13

**Authors:** Bailey E. McGuire, Andrew G. Hettle, Chelsea Vickers, Dustin T. King, David J. Vocadlo, Alisdair B. Boraston

**Affiliations:** 1 Department of Biochemistry and Microbiology, University of Victoria, Victoria, British Columbia, Canada; 2 Department of Molecular Biology and Biochemistry, Simon Fraser University, Burnaby, British Columbia, Canada; 3 Department of Chemistry, Simon Fraser University, Burnaby, British Columbia, Canada

**Keywords:** glycoside hydrolase, carrageenan, blood group antigen, galactosidase, Pseudoalteromonas, X-ray crystal structure, glycoside-hydrolase, X-ray crystallography, enzyme structure, structural biology, galactose

## Abstract

α-Linked galactose is a common carbohydrate motif in nature that is processed by a variety of glycoside hydrolases from different families. Terminal Galα1–3Gal motifs are found as a defining feature of different blood group and tissue antigens, as well as the building block of the marine algal galactan λ-carrageenan. The blood group B antigen and linear α-Gal epitope can be processed by glycoside hydrolases in family GH110, whereas the presence of genes encoding GH110 enzymes in polysaccharide utilization loci from marine bacteria suggests a role in processing λ-carrageenan. However, the structure–function relationships underpinning the α-1,3-galactosidase activity within family GH110 remain unknown. Here we focus on a GH110 enzyme (PdGH110B) from the carrageenolytic marine bacterium *Pseudoalteromonas distincta* U2A. We showed that the enzyme was active on Galα1–3Gal but not the blood group B antigen. X-ray crystal structures in complex with galactose and unhydrolyzed Galα1–3Gal revealed the parallel β-helix fold of the enzyme and the structural basis of its inverting catalytic mechanism. Moreover, an examination of the active site reveals likely adaptations that allow accommodation of fucose in blood group B active GH110 enzymes or, in the case of PdGH110, accommodation of the sulfate groups found on λ-carrageenan. Overall, this work provides insight into the first member of a predominantly marine clade of GH110 enzymes while also illuminating the structural basis of α-1,3-galactoside processing by the family as a whole.

The ABH glycan antigens define the ABO blood types, the appropriate matching of which is a key consideration in blood transfusions and organ transplantations. The O-blood group, defined by the smaller H antigen, is considered a universal donor. Accordingly, enzymatic conversion of the more elaborate and immunogenic A/B antigens to the H antigen provides an attractive route to avoid antigen mismatching and create a ready supply of universal donor blood. A campaign to identify enzymes that could hydrolyze terminal α-1,3–linked *N*-acetyl d-galactosamine and/or d-galactose from the A and B antigens, respectively, and thereby provide a set of biocatalytic tools to perform this antigen switching revealed a set of enzymes with specific B antigen *exo*-α-1,3-galactosidase activity (1, 2). These enzymes were the founding members of GH110 (glycoside hydrolase 110) family, of which all currently characterized members are *exo*-α-1,3-galactosidases that are able to hydrolyze the B antigen glycan (Galα1–3(Fucα1–2)Gal-R) (1, 2, 3). The GH110 family presently has over 400 classified members in the Carbohydrate-Active Enzyme Database (4). These putative enzymes are encoded by genes found in the genomes of a variety of environmental and host-adapted bacteria. Most recently, genes encoding proteins that are classified by amino acid sequence identity into GH110 have been identified in polysaccharide utilization loci (PULs) from human gut microbiome bacteria and marine bacteria; these PULs are postulated to target λ-carrageenan, which is a polysaccharide found in marine algae (5, 6, 7).

Most of the photosynthetically fixed carbon present on Earth is in land plants with the amount of photosynthetically fixed carbon in the oceans (*i.e.* present in microalgae and macroalgae (seaweed)) estimated to be only ∼1/200 that of terrestrial plant biomass. However, the annual turnover rate (total mass/time) of photosynthetically fixed carbon in the oceans is roughly equal to that on land, indicating a highly dynamic process with a normalized rate of recycling that is ∼2 orders of magnitude greater than on land (8). This is a remarkable biotransformation that occurs in the marine environment, and it is largely made possible by the metabolic capabilities of marine microbes, who return the carbon locked in algal storage and structural polysaccharides to the global carbon cycle (9). The dedicated metabolic pathways, which include numerous glycoside hydrolase, deployed by marine microbes to perform this biotransformation are uniquely adapted to the distinctive chemical compositions of marine algal polysaccharides. However, the identification and molecular details of several pathways that target major classes of marine polysaccharides, such as λ-carrageenan, remain to be uncovered. Understanding these pathways is key to, for example, the development of complete biogeochemical models of the global carbon cycle (10), comprehending the rise and fall of algal blooms (11), unlocking farmable seaweed biomass feedstocks for the generation of biofuels or other high-value products (12), and even identifying and engineering the metabolic capabilities of the human gut microbiome (7, 13, 14).

Carrageenans are a family of abundant marine polysaccharides comprising unbranched, high-molecular-mass sulfated galactans. They are typically found in the cell walls of marine red macroalgae, where they can make up to 50% of the dry mass. The backbone comprises d-galactose with alternating α-1,3- and β-1,4-linkages. The occurrence and specific patterns of sulfate esters on the free hydroxyl groups of the galactose backbone, along with the presence or absence of 3,6-anhydro-d-galactose, gives rise to numerous different carrageenan families (15, 16, 17). λ-Carrageenan is made of neocarrabiose motifs in which d-galactose-2,6-sulfate is α-1,3–linked to d-galactose-2-sulfate, and this disaccharide is joined by β-1,4-glycosidic linkages forming a linear λ-carrageenan polymer that is distinct from other carrageenans by its lack of 3,6-anhydro-d-galactose. Presently, how microbes process λ-carrageenan is poorly understood with only *endo*-acting β-1,4-λ-carrageenases from *Pseudoalteromonas carrageenovora* 9^T^ (18, 19) having been identified. To date, no other enzymes having activity consistent with λ-carrageenan processing have been experimentally identified, including the distinct absence of identified enzymes that are active on the α-1,3–linkages. We postulate that this is an activity performed by the GH110 enzymes found in λ-carrageenan PULs.

Toward testing this hypothesis, we characterized the structure and function of PdGH110B. This enzyme is encoded by a gene we identified in the recently reported genome of *Pseudoalteromonas distincta* U2A (referred to as U2A for brevity) (6). PdGH110B has ∼25% amino acid sequence identity with the characterized GH110 enzymes from *Bacteroides* sp. (1, 2) and ∼94% amino acid sequence identity with a putative GH110 enzyme present in the *P. carrageenovora* 9^T^ PUL that is proposed to target λ-carrageenan (5). Here we demonstrate that PdGH110B is an α-galactosidase that can hydrolyze the Galα1–3Gal disaccharide, but not the blood group B-trisaccharide [Galα1–3(Fucα1–2)Gal], thus distinguishing it from previously characterized GH110 enzymes. The structural determination of PdGH110B in complex with enzyme substrate and products revealed the parallel β-helix fold of GH110 enzymes and a −1 subsite (20) that accommodates an unmodified galactose residue. An analysis of the structure points to a potential +1′ subsite that is key to accommodating sulfate modifications, as present in λ-carrageenan, or fucose, a defining constituent of the blood group B antigen glycan. Overall, the results are consistent with PdGH110B representing the founding member of a GH110 subfamily that *exo*-hydrolytically processes terminal α-linked galactose residues in λ-carrageenan while providing general insight into the specificity of the family as a whole.

## Results

### Identification of a GH110 in Pseudoalteromonas distincta sp. U2A

U2A was isolated from the marine environment for its capacity to grow on macroalgal polysaccharides, including carrageenan, as previously described (6). We identified two adjacent genes (locus tags EU511_08545 and EU511_08540) encoding proteins with 30% sequence identity to one another and ∼25% sequence identity to previously characterized GH110 enzymes. This pair of proteins displayed 98 and 94% amino acid sequence identity to two putative orthologous GH110 enzymes in *P. carrageenovora* 9^T^ that are encoded by adjacent genes in a presumed λ-carrageenan PUL (5). A gene truncation of EU511_08540, encoding a protein we refer to as PdGH110B, lacking the predicted signal peptide was overproduced and purified, followed by qualitative assessment of activity on the synthetic substrates *p*NP-α-d-galactopyranoside and *p*NP-β-d-galactopyranoside. Recombinant PdGH110B showed activity only on *p*NP-α-d-galactopyranoside and a pH optimum of ∼5.6 (Fig. S1A).

**Figure 1:**
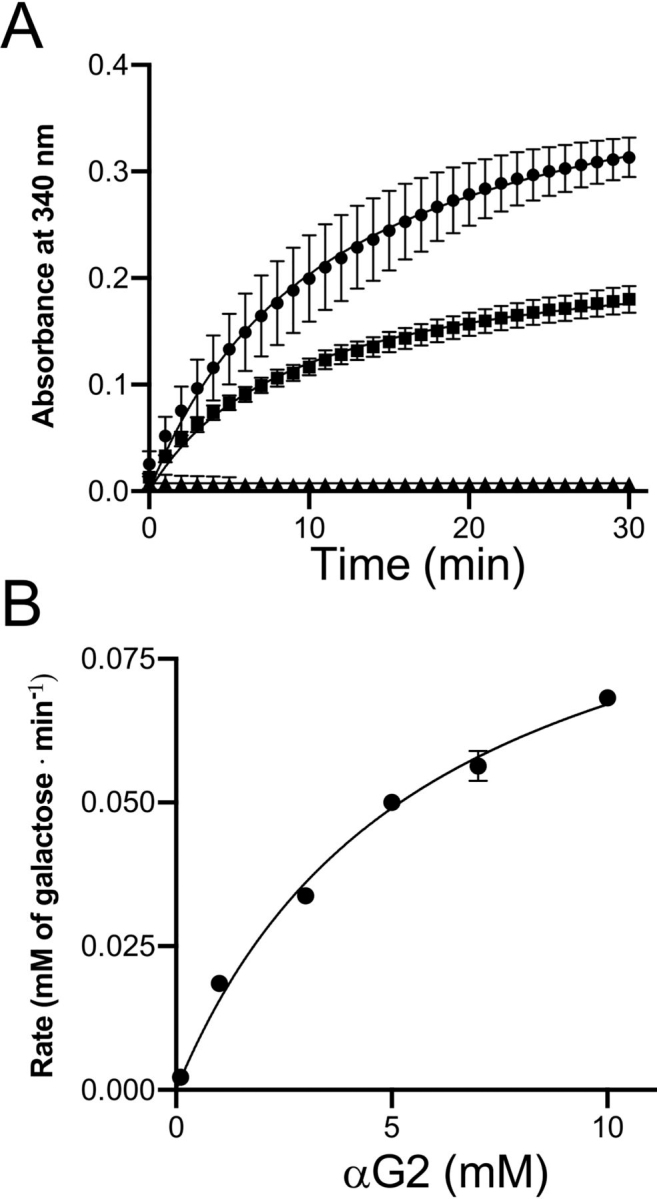
**Activity of PdGH110B on α-1,3– and β-1,4–galactobiose.***A*, galactose release curve showing release of galactose units by PdGH110B when incubated with αG2 (*closed squares*) and βG2 (*closed triangles*), along with a d-galactose standard (*closed circles*). *B*, kinetic analysis of PdGH110B activity on αG2 at 25 °C using a galactose release kit to measure units of galactose released. In both panels, *error bars* show standard deviations of measurements made in triplicate.

We further tested the activity of PdGH110B on more natural substrates using Galα1–3Gal (αG2) and Galβ1–4Gal (βG2), which represent the basic unmodified motifs present in λ-carrageenan, by quantifying galactose release. PdGH110B released d-galactose when incubated with αG2, whereas there was no activity on βG2 (Fig. 1*A*). The *K_m_* and *k*_cat_ for αG2 were 5.9 ± 1.1 mm and 18.3 ± 0.002 s^−1^, respectively (Fig. 1*B*). Given the activity of other GH110 enzymes on the blood group B glycan, we tested PdGH110B using TLC but could not detect any activity (Fig. S1B).

### The overall structure of PdGH110B α-1,3-galactosidase in complex with D-galactose

**Figure 2:**
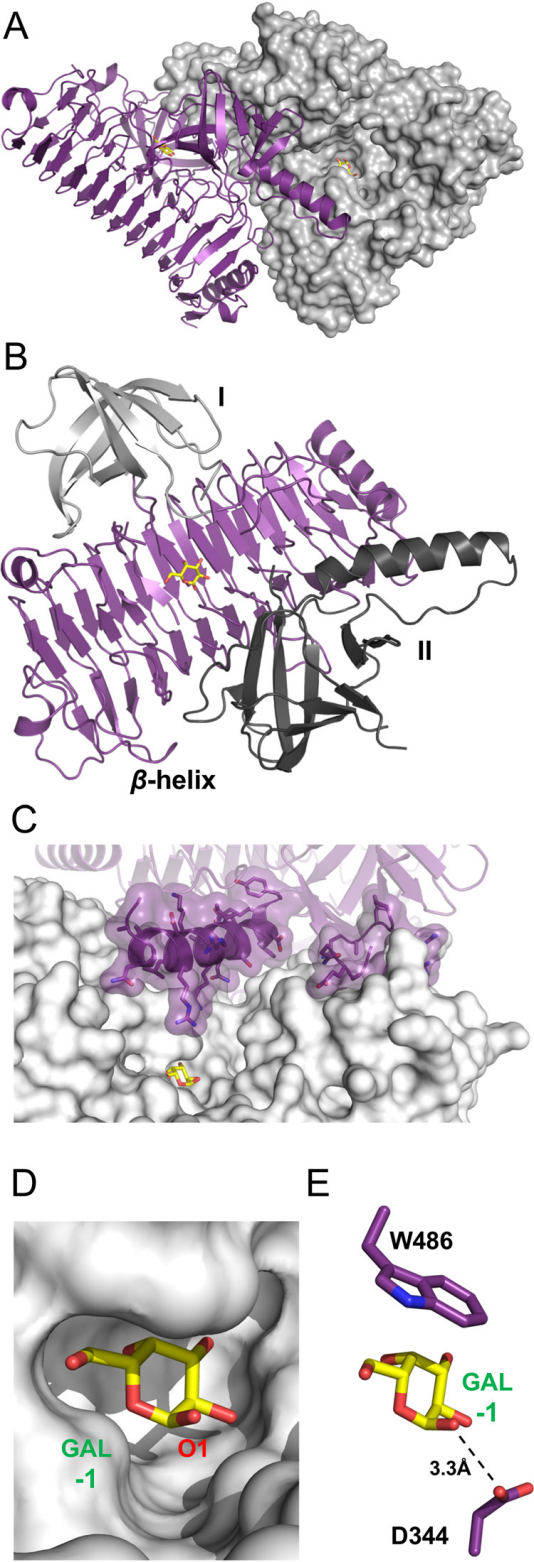
**Structural features of PdGH110B in complex with D-galactose.***A*, the structure of the PdGH110B dimer. Monomer A (*purple*) is shown in cartoon representation, and monomer B (*gray*) is shown as a solvent-accessible surface representation. *B*, cartoon representation showing the different structural domains of PdGH110B, the β-helix domain (*purple*), domain I (*gray*), and domain II (*dark gray*). *C*, the association of the domain II α-helix extending into the neighboring chain's active site. The coloring is the same as in *A*. *D*, the active site pocket, shown as a solvent-accessible surface in *gray*, sequesters the galactose residue. The O1, which would be engaged in a glycosidic linkage, is indicated. *E*, the interaction of the galactose residue with the likely catalytic acid residue. Interacting side chains are shown in *purple*, and the water molecule is shown as a *red sphere*. In all *A–E*, the D-galactose monosaccharide is represented as *yellow sticks*.

An initial preliminary structure of PdGH110B was determined by single-wavelength anomalous dispersion using a cadmium derivative. This initial model was used to solve the structure of PdGH110B in complex with a d-galactose monosaccharide to 2.35 Å resolution. The crystal structure of PdGH110B in complex with d-galactose revealed two chains in the asymmetric unit. Residues 25–238/241–616 for one molecule and residues 21–182/186–238/241–616 for the second molecule were modeled, with the missing residues residing in loop regions. The noncrystallographic dimer shows no evidence of being stable; however, each monomer in the asymmetric unit forms a crystallographic dimer (Fig. S2A). The crystallographic dimers (Fig. 2*A*) have a total molecular interface, determined by PISA (Proteins, Interfaces, Structures and Assemblies) (21) analysis, of 2300 Å^2^, predicting a stable dimeric state. Dynamic light scattering analysis of PdGH110B in solution at protein concentrations of 0.18, 0.37, and 0.73 mg/ml yielded a molecular mass of 136.3 ± 9.4 kDa. The expected molecular mass of the PdGH110B monomer is 67.9 kDa, resulting in an expected molecular mass of 135 kDa for a dimer. This indicates that PdGH110B adopts a dimeric quaternary structure, which is most likely the biologically relevant assembly.

The overall fold of PdGH110B is that of a right-handed parallel β-helix of 11 complete turns. A structural homology search using the DALI server (22) identified a fold most similar to those of a GH87 α-1,3-glucanase from *Bacillus circulans* (23), as well as two epimerases, AlgE4 and AlgE6, from *Azotobacter vinelandii* (16) (PDB code 5LW3). This core β-helix is surrounded by two small β-barrel domains (domains I and II) that contribute to the residues involved in dimerization of PdGH110B (Fig. 2*B*). The α-helix of domain II from the adjacent monomer folds over and along the wall of the active site, which was identified by a bound d-galactose, with several amino acid side chains protruding into the cleft that contains the active site pocket (Fig. 2*C*).

The bound d-galactose monosaccharide was identified by clear electron density (Fig. S2B) found in the central region of the β-helix domain of both active sites of the dimer. The modeled monosaccharide occupied a pocket that sequesters the monosaccharide in a fashion that is typical for glycoside hydrolases that are *exo*-acting on the nonreducing end of glycans (Fig. 2*D*). Specifically, Asp-344 is located within hydrogen bonding distance of the C1-OH, where the scissile bond of an intact substrate would be (Fig. 2*E*), indicating that this residue is a likely candidate to play the catalytically essential role of general acid. To trap an intact substrate complex of the enzyme, we targeted this residue to generate an inactive D344N mutant, which indeed lacked activity on *p*NP-α-galactopyranoside.

### Structure of PdGH110B in complex with α-1,3-galactobiose (αG2)

**Figure 3:**
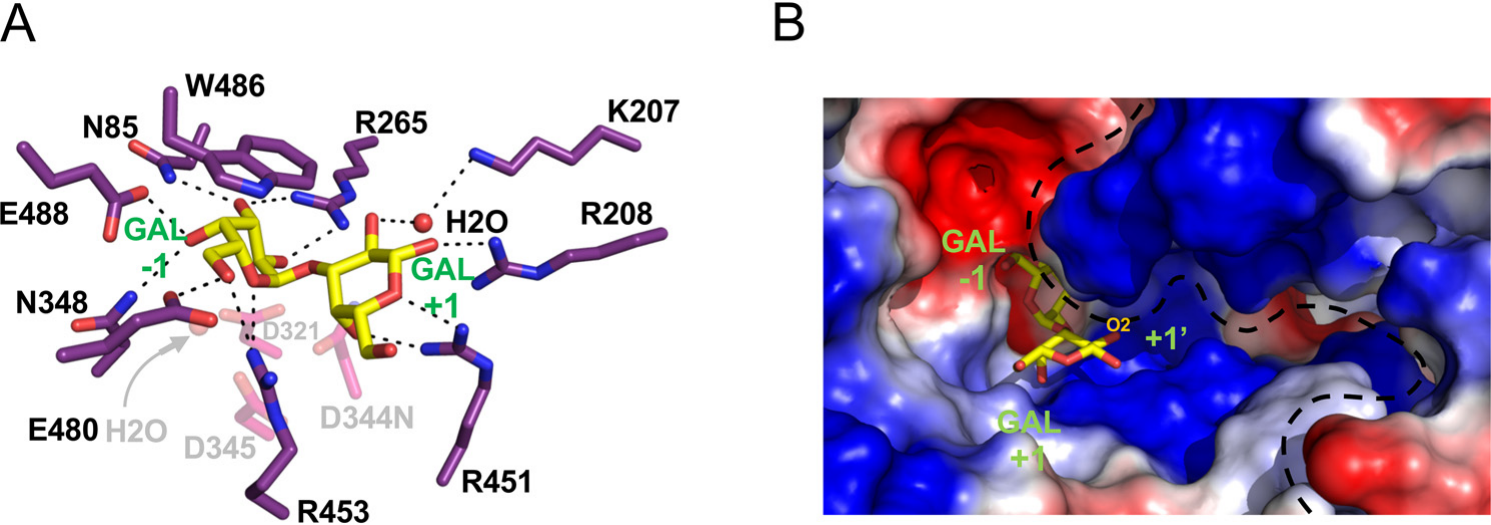
**PdGH110B D344N in complex with αG2.***A*, the specific interactions of αG2 with the PdGH110B active site. Residues forming the −1 and +1 subsites are represented as *purple sticks*, and the catalytic machinery is shown as *transparent pink sticks*. αG2 is represented as *yellow sticks*, water molecules are *red spheres*, and hydrogen bonds are shown as *dashed lines*. *B*, the solvent-accessible surface of the PdGH110B dimer shown as electrostatic potential. Positive and negative surface potential are shown in *blue* and *red*, respectively. The O_2_ that may bear a sulfate residue in λ-carrageenan is indicated. The *dashed line* defines the interface between the two monomers of the PdGH11B dimer. In both panels the active site subsites and sugar residues are indicated in *green*.

Crystals of the PdGH110B D344N mutant were soaked with an excess of αG2, and the structure was determined to 2.20 Å resolution. The refined structure revealed four monomers of PdGH110B D344N in the asymmetric unit. The monomers were organized as two noncrystallographic dimers with identical arrangements to the crystallographic dimer observed in the PdGH110B d-galactose complex, supporting the concept that the dimer is a stable quaternary structure. Clear electron density for the αG2 disaccharide was found in each monomer active site (Fig. S3) with the intact glycosidic linkage spanning the −1 subsite and +1 subsites and the catalytic machinery (Fig. 3*A*).

There is an extended network of hydrogen bonds, as well as a single interacting aromatic residue, made between the αG2 molecule and the enzyme active site (Fig. 3*A*). The C2–C6 portion of the α-face of the d-galactose unit in the −1 subsite sits on a hydrophobic platform created by Trp-486, interacting through CH–π interactions often employed by CAZymes (24). The remainder of the −1 subsite is created by an extensive hydrogen bond network comprising interactions between Asn-85, Glu-488, Asn-348, and Glu-480 and the C3-OH, C4-OH, and C6-OH hydroxyl groups. Arg-265 coordinates both C2-OH and C3-OH, and Arg-453 interacts with C6-OH and the endocyclic oxygen (Fig. 3*A*). The +1 subsite is formed exclusively by the positively charged side chains of Arg-451, Arg-208, and through a water coordinated by Lys-207. The electrostatic potential of the active site surface indicates a generally acidic −1 subsite but a +1 subsite and neighboring surfaces that are basic (Fig. 3*B*).

**Figure 4:**
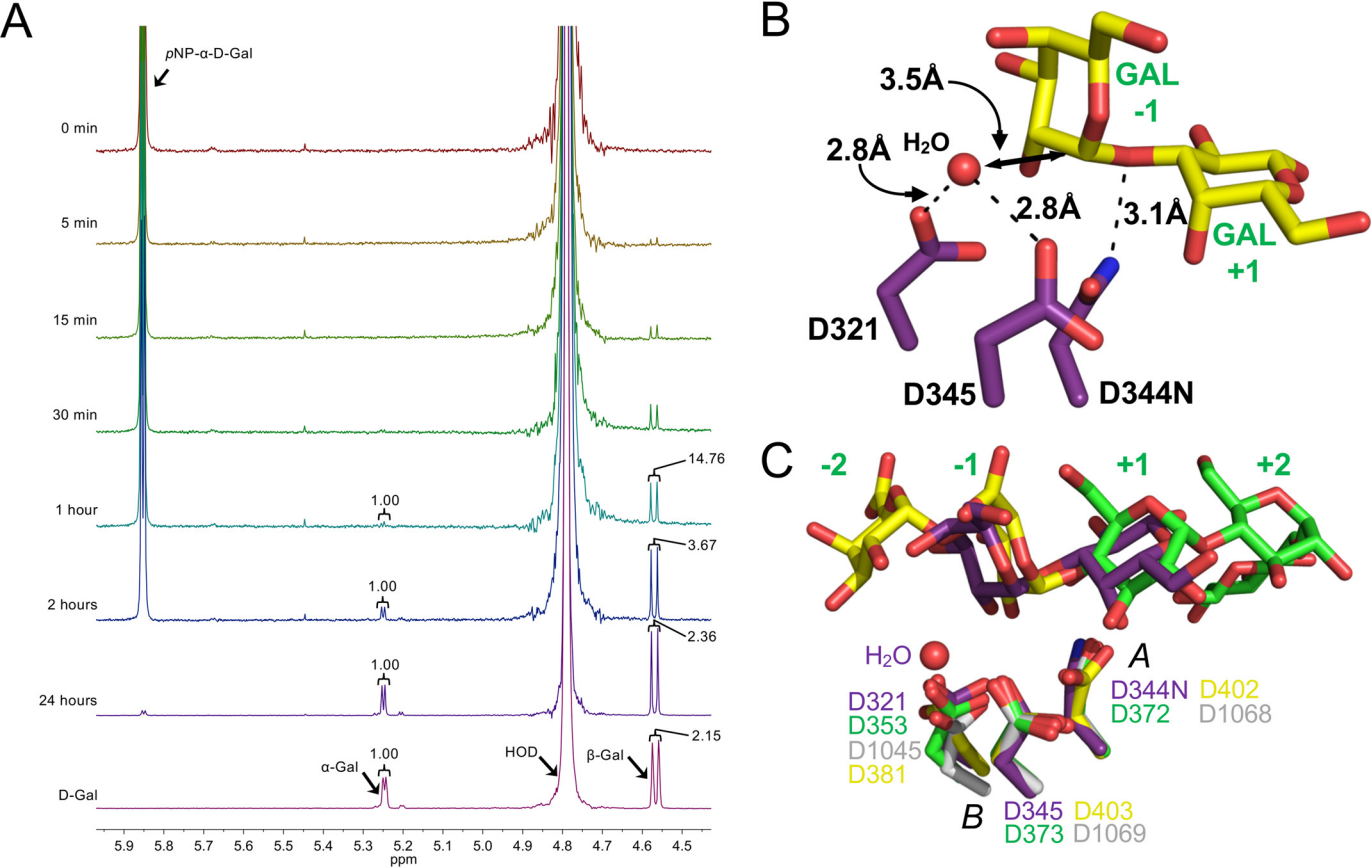
**PdGH110B using an inverting catalytic mechanism.***A*, ^1^H NMR spectra of *p*NP-α-d-galactopyranoside treated with PdGH110B measured at various time points. Signals with NMR chemical shifts corresponding with those distinctive of *p*NP-α-d-galactopyranoside, α-galactopyranoside, and β-galactopyranoside are labeled with integrated peak areas shown where applicable. *B*, αG2 (*yellow sticks*) bound in the active site of PdGH110B D344N. The three catalytic residues are represented as *purple sticks*, hydrogen bonds are *dashed lines*, and the water molecule is a *red sphere*. *C*, an overlay of the PdGH110B αG2 complex (*purple sticks*) with the GH49 isopullulanase from *Aspergillus niger* (*green*; PDB code 2z8g) (34), the GH28 exo-polygalacturonase from *Yersinia enterocolitica* (*yellow*; PDB code 2uvf) (36), and the GH87 α-1,3-glucanase from *Paenibacillus glycanilyticus* (*gray*; PDB code 6k0n) (35). The active site subsites are labeled in *green*. The putative acid is indicated with *A*, and the pair of putative bases is indicated with *B*.

GH110 enzymes were previously shown to operate through use of a single displacement, or inverting, catalytic mechanism (1). We confirmed this for PdGH110B by using ^1^H NMR to monitor the initial release of the β-anomer of d-galactose from *p*NP-α-d-galactopyranoside, which indicates inversion of the anomeric configuration of C1 involved in the glycosidic bond (Fig. 4*A*). The architecture of the PdGH110B catalytic center is also consistent with an inverting catalytic mechanism (25, 26) (Fig. 4*B*). In the αG2 complex with the mutant enzyme, Asn-344, which would be Asp-344 in the WT enzyme, is 3.1 Å from the glycosidic oxygen and appropriately positioned to act as a general acid. A water molecule that sits 3.5 Å beneath C1 of the d-galactose residue in the −1 subsite is suitably positioned to be activated as a nucleophile by Asp-321 and/or Asp-345 (Fig. 4*B*).

## Discussion

The GH110 family was initially identified by examination of members that specifically removed the immunodominant α-1,3–linked galactose residues of blood group B antigen. Subsequent characterization of additional GH110 enzymes re-vealed some to be less stringent by possessing the ability to process the linear α-Gal epitope, as well as the blood group B antigen. In contrast to the majority of other α-galactosidases, which employ a retaining catalytic mechanism (*i.e.* GH4, GH27, GH31, GH36, GH57, and GH97 (27, 28, 29, 30, 31, 32)), family GH110 was shown to utilize an inverting mechanism for this hydrolytic activity. Here, through examination of PdGH110B, which originates from a marine bacterium and has relatively low amino acid sequence identity to previously characterized GH110 members, we also demonstrated α-Gal activity, although it differed from other GH110 enzymes by its inability to hydrolyze the blood group B antigen. The structure of PdGH110B revealed the molecular basis of the inverting mechanism utilized by the family. Notably, the core parallel β-helix fold and catalytic machinery of PdGH110 is conserved within GH families 28, 49, and 87 (Fig. 4*C*) (33, 34, 35). GH28 and GH49 are classified into glycoside hydrolase clan GH-N. Given that the fold and catalytic machinery of these founding families of the clan are conserved with GH87 and GH110, we note that it is likely that the latter two GH families also belong to clan GH-N.

**Figure 5:**
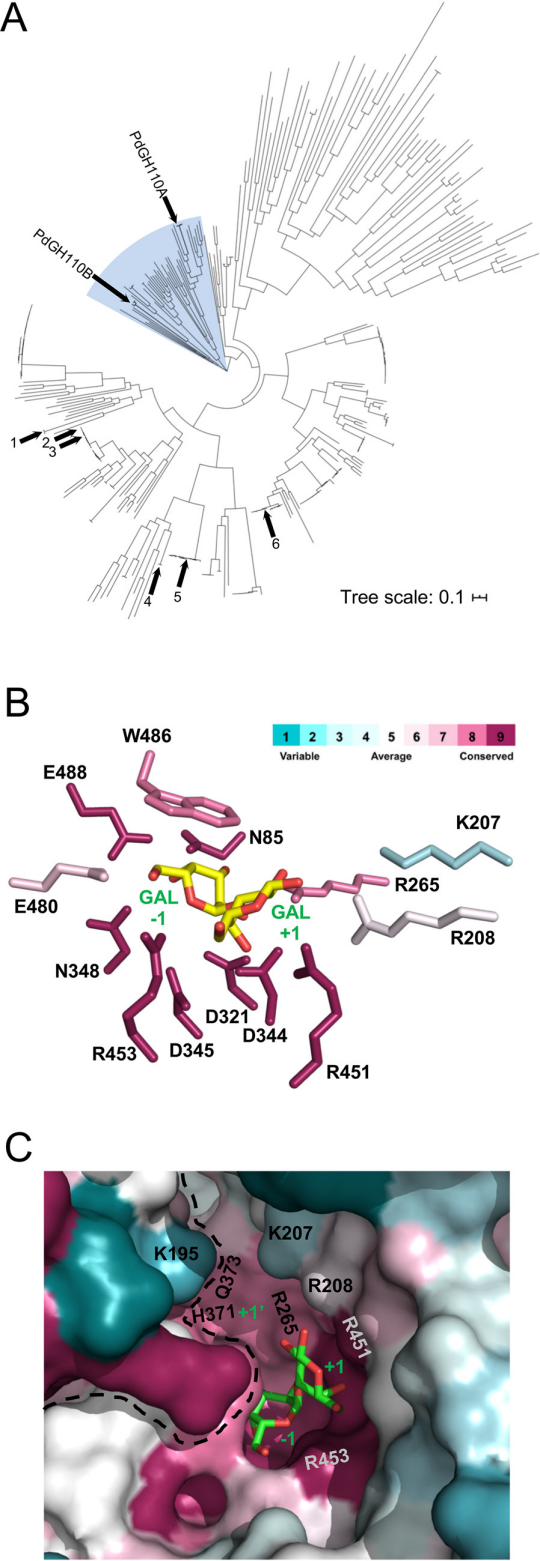
**Phylogeny and conservation of glycoside hydrolase family 110 sequences.***A*, phylogenetic tree of family GH110 constructed from 334 sequences extracted from the CAZy database. The numbered arms are as follows: *arm 1*, BtGal110A from *Bacteroides thetaiotaomicron*; *arm 2*, BtGal110B from *B. thetaiotaomicron*; *arm 3*, BfGalA from *Bacteroides fragilis*; *arm 4*, SaGal110A from *Streptomyces avermitilis*; *arm 5*, BbAgaBb from *Bifidobacterium bifidum*; and *arm 6*, BfGal110B from *B. fragilis.* The *shaded region* indicates the “marine” clade. See also Fig. S4 and Table S1 for complete annotation of the tree. *B*, the conservation of active site residues of GH110 mapped onto the structure of PdGH110B by ConSurf analysis (46). *C*, conservation of the active site shown as accessible surface representation. The color scheme representing degree of residue conservation in *B* and *C* is shown in *B*. Active site subsites and sugar residues are labeled in *green*.

A phylogenetic tree constructed from 334 GH110 amino acid sequences, including PdGH110B and another from U2A (PdGH110A), displays several distinct clades (Fig. 5*A* and Fig. S4). Notably, the sequences originating primarily from marine microbes form their own clade that branches off directly from the origin, hinting at evolution toward potentially distinct functions. Indeed, the *P. carrageenovora* 9^T^ orthologue of PdGH110B (∼94% amino acid sequence identity) resides in a locus proposed to target the highly sulfated marine galactan λ-carrageenan (5). The genes neighboring that encoding PdGH110B in U2A show similarly high amino acid sequence identity to components of the putative *P. carrageenovora* 9^T^ λ-carrageenan PUL. This led to the hypothesis that PdGH110B (and its orthologue) would target λ-carrageenan. The activity of PdGH110B on αG2, and the molecular recognition thereof, is consistent with *exo*-α-1,3-galactosidase activity on the nonreducing ends of λ-carrageenan. In contrast, because of its ^1^C_4_ chair conformation, the 3,6-anhydro-d-galactose residue found at the nonreducing termini of κ-and ι-neocarrageenoligosaccharides would not be accommodated in the active site of PdGH110B. Notably, the architecture of the −1 subsite leaves no room for a sulfate modification on either C2 or C6 of the galactose residue, indicating that PdGH110B requires an unmodified d-galactose at the nonreducing end of its substrate. This is consistent with our inability to observe activity for this enzyme on sulfated λ-neocarrageenoligosaccharides (not shown) and suggests that preprocessing of λ-carrageenan by as-yet-unidentified sulfatases would be necessary prior to the action of PdGH110B.

An examination of amino acid conservation within the −1 subsite of the representative GH110 sequences reveals this subsite to be remarkably conserved across the family (Fig. 5*B*), including among the characterized enzymes (Fig. S5), suggesting that *exo*-α-galactosidase activity is a general feature of the family. This observation points to features of the plus (+) subsites as being critical determinants of substrate selectivity. In the case of PdGH110B, a series of specific interactions, particularly those mediated by Arg-451, select for a galacto-configured sugar in the +1 subsite. In λ-carrageenan, the galactose residue in this subsite would likely bear a 2-sulfate group. The structure of PdGH110B in complex with αG2 shows a small pocket, which we will refer to as a +1′ subsite, with the appropriate size and charge to accommodate a sulfate modification (Fig. 3*B*). Although PdGH110B was active on αG2 (*i.e.* lacking sulfates) the high *K_m_* (∼5 mm) suggests nonoptimal recognition of this substrate; 2-sulfation of the galactose in the +1 subsite may be expected to improve binding of the substrate. Thus, we suggest that the features of the PdGH110B active site are entirely consistent with λ-carrageenan recognition.

The +1 subsite has one conserved residue, Arg-451, which makes specific interactions with the galactose residue in the +1 subsite that are likely maintained throughout the family (Figs. 3*A* and 5*B*). The other residue in this subsite that makes a direct interaction, Arg-208, is not well-conserved (Fig. 5*B*). Nevertheless, the noteworthy conservation of the −1 subsite and conservation of Arg-452 in the +1 subsite, which makes directional interactions specific to a galacto-configured monosaccharide, reveal a mechanism that is shared by the GH110 family to recognize a Galα1–3Gal motif. Lys-207 makes potential water-mediated hydrogen bond to the galactose in the +1 subsite; however, it appears more relevant to the +1′ subsite of PdGH110B, which is not well-conserved among the GH110 family (Fig. 5, *B* and *C*). In particular, Lys-195 (contributed by the other monomer of the dimer) and Lys-207 on either side of the +1′ subsite close it off this subsite in PdGH110B making it of insufficient size to accommodate the 2-fucosyl residue of the blood group B antigen. These residues are not well-conserved among the family as a whole (Fig. 5*C*) nor in the characterized enzymes (Fig. S5). In the characterized enzymes, Lys-195 resides in a variable region, which in PdGH110B comprises the “finger” that extends between the two monomers of the dimer (Fig. 2*C* and Fig. S5), although Lys-207 is typically an amino acid with a smaller sidechain (Fig. S5). These alterations seen other GH110 enzymes likely open up the +1′ subsite, allowing it to accommodate the 2-fucosyl residue of the blood group B antigen. Furthermore, these structural changes, along with substitutions of the poorly conserved Arg-208, would likely reduce the basic nature of the +1′ subsite, making it less suitable for accommodating an anionic sulfate residue and more appropriate for a neutral fucosyl residue.

To date, all characterized examples of GH110 enzymes display α-1,3-galactosidase activity. These enzymes partition into three classes of activity: enzymes that are specific for the blood group B antigen, those that are active on both the blood group B antigen and linear α-1,3-galactose epitopes, and now a class represented by PdGH110B that is selective for linear α-1,3–linked galactose. PdGH110B notably sorts into a clade that comprises proteins from primarily marine microbes. This observation likely reflects that enzymes in this clade have adapted to enable biological processing of λ-carrageenan. Indeed, the other entries in this clade that are not from marine microbes are typically from human gut bacteria, which themselves may also be able to target λ-carrageenan present in the diet. Further examination of this marine clade of GH110 enzymes may ultimately reveal additional adaptations that confer the ability to accommodate, or even specifically recognize, the sulfate modifications on λ-carrageenan.

## Experimental procedures

### Materials

All reagents, chemicals and other carbohydrates were purchased from Sigma unless otherwise specified.

### Cloning and mutagenesis

The gene fragment encoding GH110B without predicted signal peptides (amino acids 24–620) was amplified from *Pseudoalteromonas distincta* U2A genomic DNA using the oligonucleotide primers 5′-CTA GCT AGC AAT GAT AAA GTG ATA GAT G-3′ (PdGH110B_fwd) and 5′-CCG CTC GAG TTA GTT TTT AGC TCT TTT AT-3′ (PdGH110B_rev). The product was ligated into pET28a between the NheI and XhoI restriction sites (underlined in sequences).

All DNA amplifications were done using CloneAmp HiFi PCR premix. Mutations were created by site-directed mutagenesis (QuikChange site-directed mutagenesis kit) using the primers 5′-CTC CAT GGA TGT TAG TAG CAT CAT TCT TTT GAC TTT CGA ATA AGT TAT C-3′ and 5′-GAT AAC TTA TTC GAA AGT CAA AAG AAT GAT GCT ACT AAC ATC CAT GGA G-3′. All constructs were sequence confirmed by bidirectional sequencing.

### Protein expression and purification

The expression plasmid of the PdGH110B was transformed into *Escherichia coli* BL21 (DE3) Star and grown in 2-liter cultures of LB broth containing 50 μg ml^−1^ kanamycin sulfate at 37 °C with agitation at 180 rpm until cell density reached an *A*_600_ of ∼0.5, at which time the temperature was dropped to 16 °C, and recombinant protein production was induced with a final concentration of 0.5 mm isopropyl β-d-thiogalactopyranoside and allowed to incubate for an additional 16 h.

The cultures were then centrifuged at 6300 × *g* for 10 min. Harvested cells were chemically lysed by resuspension in 35% (w/v) sucrose, 1% (w/v) deoxycholate, 1% Triton X-100, 500 mm NaCl, 10% glycerol, 20 mm Tris (pH 8.0), 10 mg lysozyme, and 0.2 μg ml^−1^ DNase. The resulting lysates were clarified by centrifugation at 16,500 × *g* for 30 min.

PdGH110B protein was purified by applying the clarified lysate supernatant to a nickel-affinity chromatography column and eluted with 20 mm Tris-HCl (pH 8.0), 500 mm NaCl, with a stepwise increase in imidazole concentration of 5, 10, 15, 20, 40, 50, 100, and 500 mm. All samples containing the protein of interest as judged by SDS-PAGE (SDS-PAGE) were concentrated with an Amicon ultrafiltration cell (EMD Millipore) with a 10-kDa molecular-mass cutoff. Proteins of interest were further purified using a HiPrep 16/60 Sephacryl S-300 HR size-exclusion chromatography column in 20 mm Tris (pH 8.0) and 500 mm NaCl.

### pH studies

The reactions with synthetic substrates were set up in triplicate using a 96-well plate and incubated at 25 °C in the dark for 1 h. The reactions (100 μl) contained 50 mm McIlvaine buffer (pH 2.9–9.6), 1 mm 1,4-dithio-d-threitol in binding buffer with 7.5% glycerol, 1 mm *p*NP-α-d-galactopyranoside or *p*NP-β-d-galactopyranoside in dH_2_O, and 1 μm recombinant PdGH110B. The reactions were stopped with an equal volume of 100 mm sodium hydroxide. The plate was read at 25 °C and 405 nm with five technical replicates, using a Molecular Devices Spectramax Plus plate reader.

### Enzyme activity measuring galactose release

The activity of recombinant PdGH110B against αG2 and βG2 was tested using the Megazyme l-arabinose/d-galactose (Rapid) assay kit (Megazyme) to detect galactose release via the oxidation of β-d-galactose and the reduction of NAD^+^ to NADH by a galactose dehydrogenase. The assays were performed in 83 mm HEPES (pH 7.5), 10 μl of kit solution 2 (NAD^+^), 2 μl of kit suspension 3 (dehydrogenase and mutarotase), 4 μg of αG2, βG2, or d-galactose in binding buffer, and 0.81 μm PdGH110B. The reactions were set up in triplicate, blanked before the addition of the kit enzymes (suspension 3), and read after addition of the enzymes every 30 s for 30 min at 25 °C and 340 nm, using a Molecular Devices Spectramax Plus plate reader.

### ^1^H NMR

Recombinant PdGH110B (1.3 ml at 1 mg/ml) was buffer-exchanged overnight at 4 °C in 1 liter of 50 mm sodium phosphate buffer (pH 5.8). PdGH110B was then buffer-exchanged into NMR buffer (50 mm sodium phosphate (pH 5.9) in 99.9% D_2_O) through dilution and reconcentration in an Amicon centrifugal filtration unit with a 10-kDa cutoff. The reaction contained 8.3 mm *p*NP-α-d-galactopyranoside and 2.9 μm recombinant PdGH110B in NMR buffer. A 10 mm d-galactose control reaction in NMR buffer was also monitored as a standard. The reactions were measured before the addition of PdGH110B and after subsequent incubations at the 5-min, 15-min, 30-min, 1-h, 2-h, and 24-h time points using a Bruker Avance II 500 MHz NMR spectrometer and a 5-mm TXI inverse probe. The data were processed using the MestReNova 10 software package.

### Phylogenetic analysis

The sequences from the glycoside hydrolase family 110 were retrieved. Of the 417 sequences, only 332 had entries in the NCBI database. A multiple sequence alignment was performed using Clustal Omega (36). The evolutionary relationships of PdGH110A, PdGH110B, and the other GH110 sequences was inferred by maximum likelihood method based on the Jones-Taylor_thornton matrix-based model using FastTree (37). The phylogenetic tree was visualized and annotated using the iTol web tool.

### Crystallization, diffraction data collection, and processing

All crystals were grown at 18 °C by hanging-drop vapor diffusion with 1:1 ratios of crystallization solution and protein. A native PdGH110B crystal grown in 0.5 m NaI, 2% Tacsimate, 0.1 m HEPES (pH 7.5), and 17% PEG 3350 was soaked for 10 min in this mother liquor containing 1 mm CdCl_2_ prior to data collection. To obtain the product complex, a PdGH110B crystal grown 0.1 m HEPES (pH 7.5), 9% PEG 6000, and 5.5% 2-methyl-2,4-pentanediol was soaked in mother liquor containing excess d-galactose prior to data collection. To obtain a substrate complex, PdGH110B_D344N was crystallized in 1 m NaI, 2% Tacsimate, 0.1 m HEPES (pH 7.5), and 17% PEG 3350 and then soaked in this mother liquor containing excess α-1,3-galactobiose prior to data collection. 25% (v/v) ethylene glycol was used as the cryo-protectant for all crystals, with the exception of the d-galactose–soaked crystal. The 2-methyl-2,4-pentanediol content of the d-galactose–soaked crystal was increased from 5.5% (mother liquor concentration) to 20% for cryo-protection.

Diffraction data were collected on an instrument comprising a Pilatus 200K 2D detector coupled to a MicroMax-007HF X-ray generator with a VariMaxTM-HF ArcSec confocal optical system and an Oxford Cryostream 800. The data were integrated, scaled, and merged using HKL2000. The data processing statistics are shown in Table 1.

**Table 1:** X-ray data collection and structure refinement statistics

	PdGH110B		PdGH110B_D344N
	Cadmium derivative	Galactose complex	α-1,3-Galactobiose (αG2) complex
**Data collection**			
Beamline	In-house	In-house	In-house
Wavelength	1.514	1.514	1.514
Space group	P2_1_	C2	P2_1_
Cell dimensions			
*a*	96.70	169.79	98.52
*b*	124.95	128.09	124.76
*c*	142.34	100.14	142.42
β	94.47	123.15	93.86
Resolution (Å)	25.00–2.00 (2.03–2.00)	30.00–2.35 (2.39–2.35)	30.00–2.20 (2.24–2.20)
*R*_merge_	0.166 (0.914)	0.127 (0.916)	0.142 (0.590)
*R*_pim_	0.040 (0.337)	0.071 (0.327)	0.096 (0.421)
CC_1/2_	0.997 (0.782)	0.989 (0.888)	0.991 (0.833)
<*I*/σ*I*>	17.9 (2.3)	10.4 (2.3)	7.1 (2.3)
Completeness (%)	99.9 (99.7)	99.6 (98.5)	98.6 (84.6)
Redundancy	17.5 (8.0)	4.0 (4.0)	3.6 (3.4)
No. of reflections	3,964,261	289,894	630,568
No. of unique reflections	89,189	17,427	174,470
**Refinement**			
Resolution (Å)		2.35	2.20
*R*_work_/*R*_free_		0.185/0.231	0.205/0.238
No. of atoms			
Protein		4663 (A), 4640 (B)	4634 (A), 4613 (B), 4625 (C), 4603 (D)
Ligand		24 (GLA), 12 (GAL)	92 (αG2)
Water		901	1661
*B*-factors			
Protein		33.9 (A), 35.1 (B)	33.7 (A), 35.2 (B), 35.2 (C), 33.5 (D)
Ligand		28.2 (GLA),54.0 (GAL)	30.0 (αG2)
Water		40.7	38.5
Root-mean-square deviation			
Bond lengths (Å)		0.007	0.002
Bond angles (°)		0.873	0.473
Ramachandran (%)			
Preferred		95.2	94.5
Allowed		4.8	5.3
Disallowed		0.0	0.2

### Structure solution and refinement

The structure of PdGH110B was determined by the single-wavelength anomalous dispersion method using the cadmium derivative. Initial phases were determined using the SHARP/autoSHARP pipeline (38). Phases were improved using PARROT (39) to perform density modification and noncrystallographic averaging. An initial model comprising ∼95% completeness was constructed by autobuilding using ARP/wARP (40). The most complete monomer from this initial model was used as a molecular replacement model to determine the structure of the PdGH110B galactose complex, which was finished by manual building with COOT (41) and refinement with REFMAC (42). A monomer from this model was then used to solve the structure of the PdGH110B_D234N structure in complex with αG2 by molecular replacement using PHASER (43) and the same building and refinement procedures.

For all structures, the addition of water molecules was performed in COOT with FINDWATERS and manually checked after refinement. In all data sets, refinement procedures were monitored by flagging 5% of all observations as “free” (44). Model validation was performed with MOLPROBITY (45). The model refinement statistics are shown in Table 1.

## Data availability

The atomic coordinates for the two crystal structures reported here have been deposited in the Research Collaboratory for Structural Bioinformatics Protein Data Bank under accession codes 7JW4 and 7JWF. All other data are available in the article and in the supporting information.
