# Supplementary material for: The structure of a family 110 glycoside hydrolase provides insight into the hydrolysis of α-1,3-galactosidic linkages in λ-carrageenan and blood group antigens
Source: J Biol Chem. 2021 Jan 13;295(52):18426–35. doi: 10.1074/jbc.RA120.015776 (PMC7939477; doi:10.1074/jbc.RA120.015776)
Supplement: Supplementary file 1 [file mmc1.pdf]

Supporting Information

The structure of a family 110 glycoside hydrolase provides insight into the hydrolysis of α-(1,3)-galactosidic linkages in λ-carrageenan and blood group antigens.

Bailey E. McGuire[1#], Andrew G. Hettle[1#], Chelsea Vickers[1,4#], Dustin T. King[2], David J. Vocadlo[2,3], and Alisdair B. Boraston[1]*.

[1]Department of Biochemistry and Microbiology, University of Victoria, PO Box 1700 STN CSC, Victoria, British Columbia, V8W 2Y2, Canada. [2]Department of Molecular Biology and Biochemistry, Simon Fraser University, Burnaby, British Columbia V5A 1S6, Canada. [3]Department of Chemistry, Simon Fraser University, Burnaby, British Columbia V5A 1S6, Canada. [4]Current address: School of Biological Sciences, Victoria University, PO Box 600, Wellington 6012, New Zealand

Running title: *Structural analysis of GH110*

[#]These authors contributed equally to this work

[*]To whom correspondence should be addressed: Alisdair B. Boraston, Department of Biochemistry and Microbiology, University of Victoria, Victoria, British Columbia, Canada, V8P 5C2; boraston@uvic.ca; Tel. +1 (250) 472-4168; Fax. +1 (250) 721-8855

Supplementary Figures

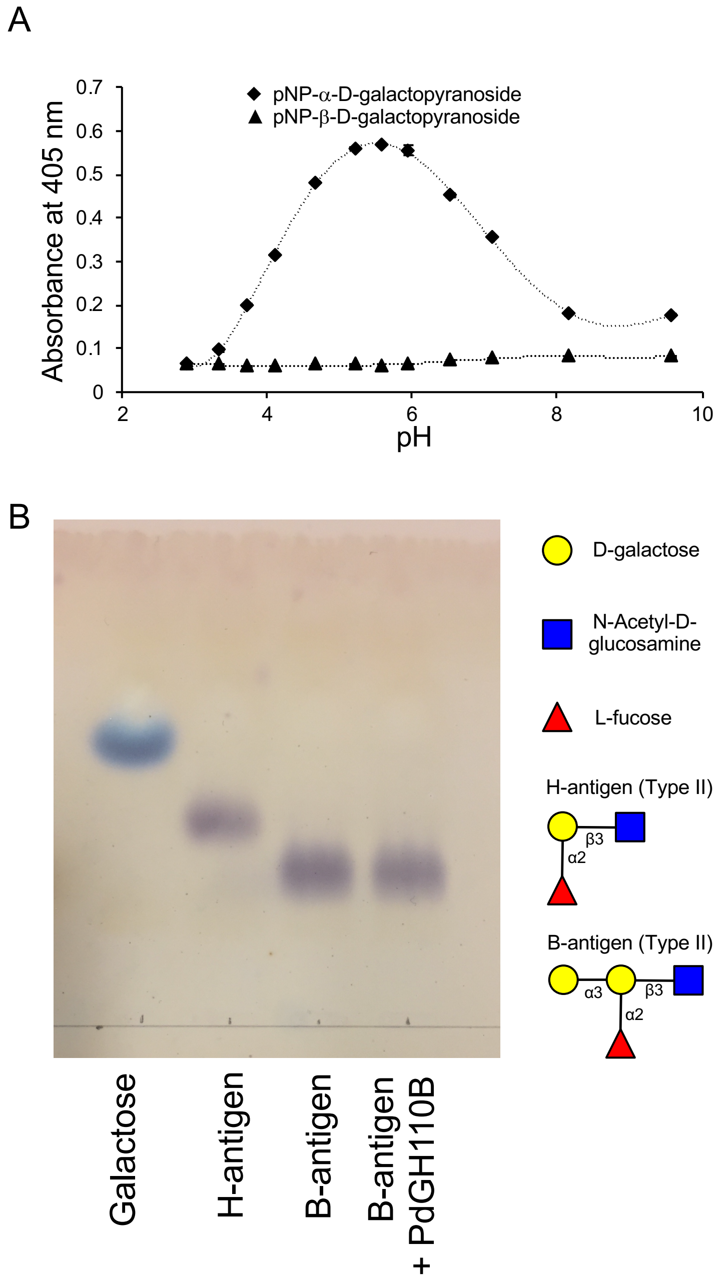

Figure S1. Activity of PdGH110B. (A) The pH optimum of PdGH110B was determined using McIlvaine buffers and either pNP-α-D-galactopyranoside or pNP-β-D-galactopyranoside. Reactions were incubated at 25 °C for 1 hour and stopped by the addition of NaOH. Data shown are the mean of three triplicates and error bars are within the size of the symbols. (B) Thin-layer

chromatography showing the lack of PdGH110 activity on the B-antigen (Type II B-tetrasaccharide) after 1 hour treatment at room temperature.

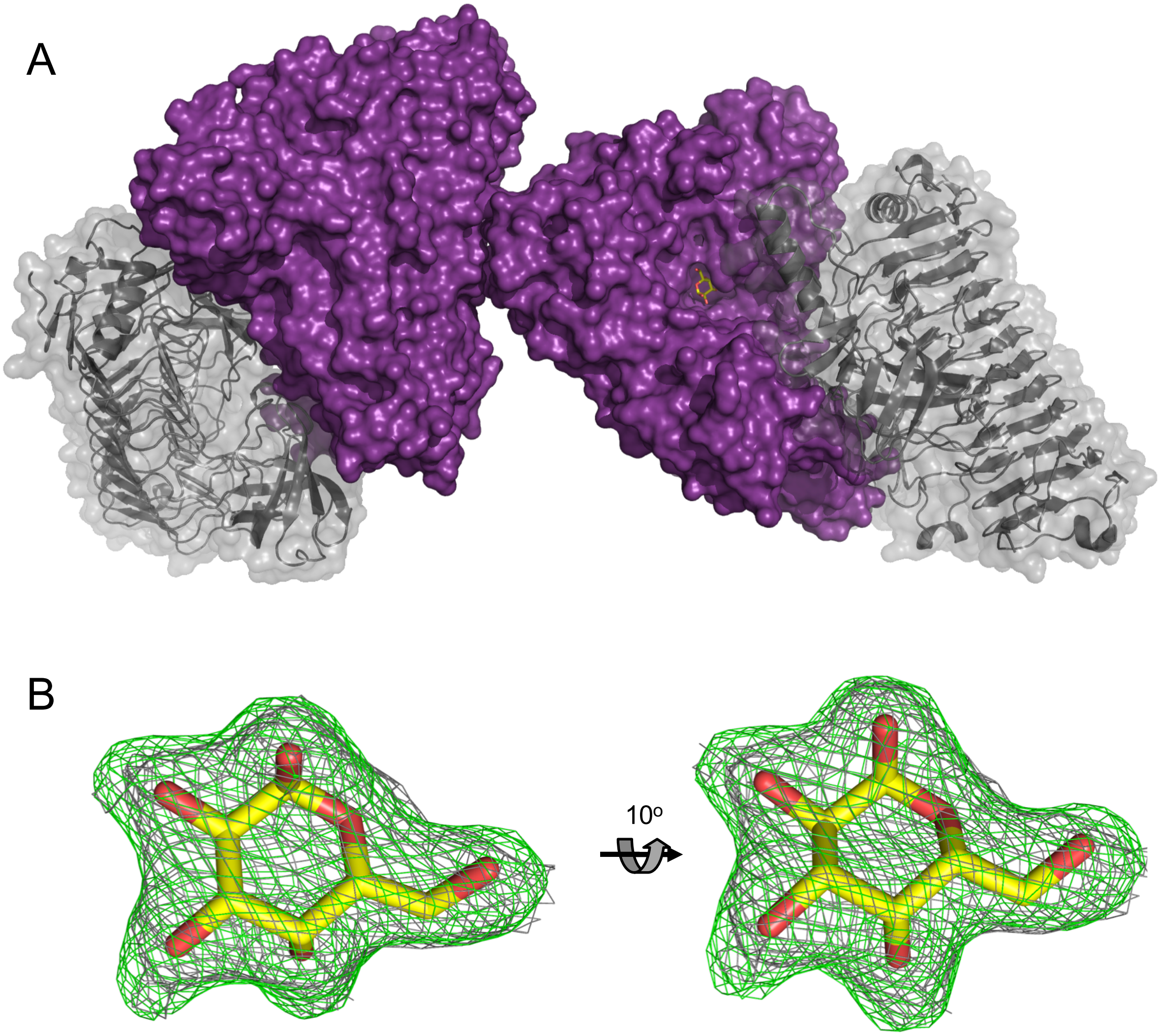

Figure S2. (A) X-ray crystal structure of the PdGH110B crystallographic dimer in complex with D-galactose. Solvent accessible surface representation of the chains in the asymmetric unit in purple, symmetry mates in grey cartoon representation with transparent solvent accessible surface. (B) Representative electron density for D-galactose modeled into the active site. The grey mesh shows the electron density map as a maximum likelihood/σ_a-weighted $2F_c$-F_o map contoured at 1.0 σ. The green mesh shows the electron density map as a maximum likelihood/σ_a-weighted F_c-F_o map (contoured at 3.0 σ) produced by refinement with the D-galactose atoms omitted. In both panels (A) and (B) D-galactose is represented as yellow sticks.

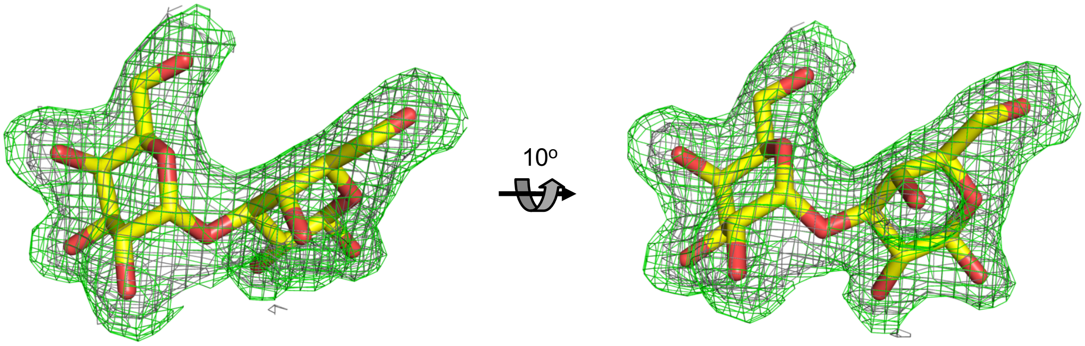

Figure S3. Representative electron density for αG2 modeled into the active site. The grey mesh shows the electron density map as a maximum likelihood/σ_a-weighted $2F_c$-F_o map contoured at 1.0 σ. The green mesh shows the electron density map as a maximum likelihood/σ_a-weighted F_c-F_o map (contoured at 3.0 σ) produced by refinement with the αG2 atoms omitted. In both panels (A) and (B) αG2 is represented as yellow sticks.

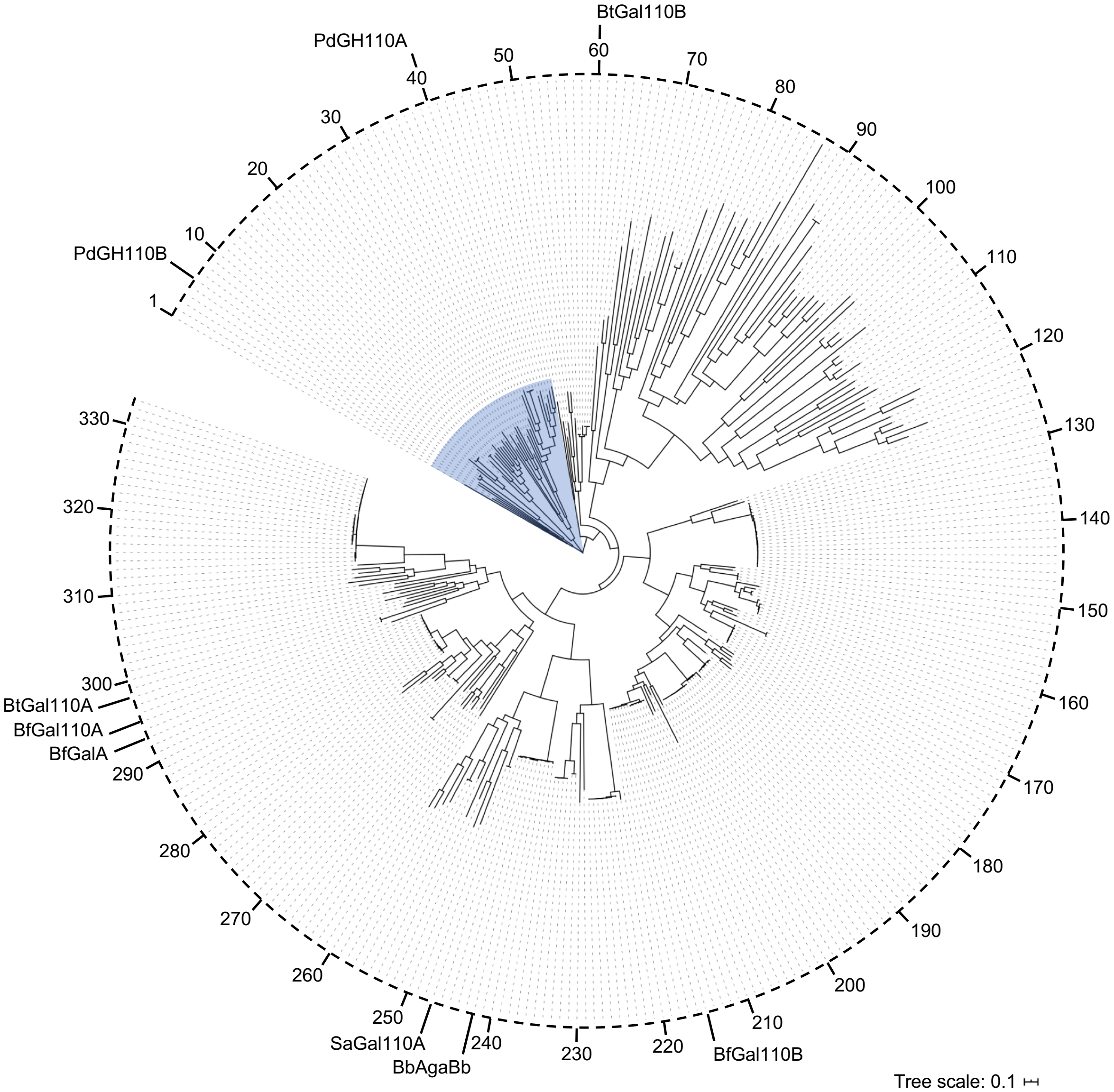

Figure S4. Phylogenetic tree of glycoside hydrolase family 110 sequences constructed from 334 sequences extracted from the CAZy database. The labeled arms are as follows: PdGH110A and PdGH110B from *Pseudoalteromonas distincta* U2A; BtGal110A from *Bacteroides thetaiotaomicron*; BtGal110B from *B. thetaiotaomicron*; BfGalA from *B. fragilis*; SaGal110A from *Streptomyces avermitilis*; BbAgaBb from *Bifidobacterium bifidum*; BfGal110B from *B. fragilis.* The blue shaded area highlights the primarily marine organism clade. See Table S1 for complete annotation of the tree.

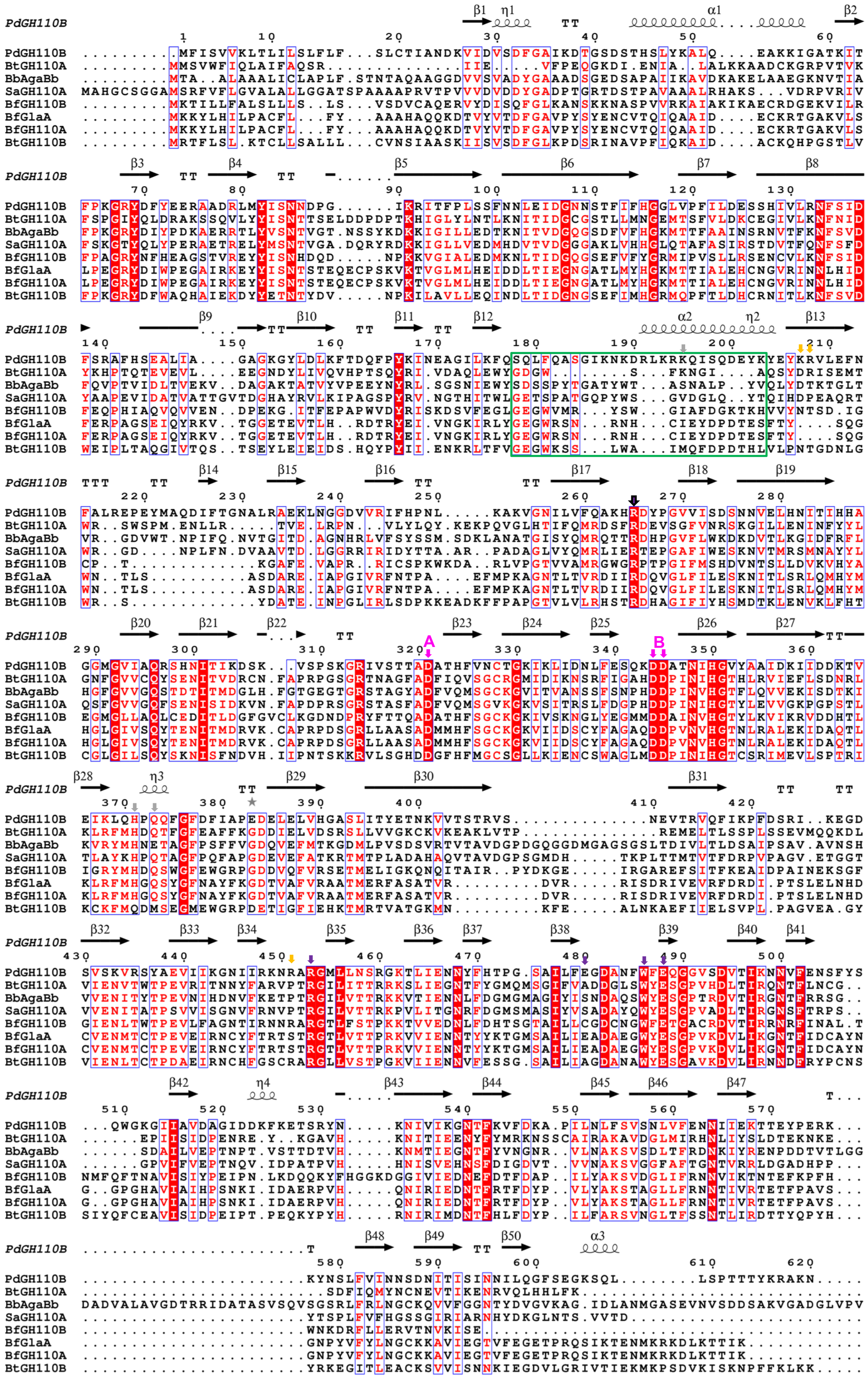
PdGH110B .........MFISVVKLTLILLSLFLF...SLCTIANDKVIDVSDFGAIKDTGSDSTHSLYKALQ....EAKKIGATKIT
BtGH110A .........MMSVWFIQLAIFAQSR.............IIE.....VFPEQGKDI.ENIA..LALKKAADCKGRPVTVK
BbAgaBb .........MTA..ALAAALICLAPLF.STNTAQAAGGDVVSVADYGAAADSGEDSAPAIIKAVDKAKELAAEGKNVTIA
SaGH110A MAHGCSGGAMSRFVFLGVALALLGGATSPAAAAPRVTPVVVDVDDYGADPTGRTDSTPAVAAALRHAKS...VDRPVRIV
BfGH110B .........MKTILLFALSLLS..LS...VSDVCAQERVYDISQFGLKANSKKNASPVVRKAIAKIKAECRDGEKVILR
BfGlaA .........MKKYLHILPACFL...FY...AAAHAQQKDTVYVTDFGAVPYSYENCVTQIQAAID....ECKRTGAKVLS
BfGH110A .........MKKYLHILPACFL...FY...AAAHAQQKDTVYVTDFGAVPYSYENCVTQIQAAID....ECKRTGAKVLS
BtGH110B .........MRTFLSL.KTCLLSALLL...CVNSIAASKIISVSDFGLKPDSRINAVPFIQKAID....ACKQHPGSTLV
PdGH110B FPKGRYDFYEERAADRLMYISNNDPG......IKRITFPLSSFNNLEIDGNNSTFIFHGGLVPFILDESSHIVLRNFSID
BtGH110A FSPGIYQLDRAKSSQVLYYISNTTSELDDPDPTKHIGLYLNTLKNITIDGCGSTLLMNGEMTSFVLDKCEGIVLKNFNID
BbAgaBb FPKGRYDIYPDKAERRTLYVSNTVGT.NSSYKDKKIGILLEDTKNITVDGQGSDFVFHGKMTTFAAINSRNVTFKNFSVD
SaGH110A FSKGTYQLYPERAETRELYMSNTVGA.DQRYRDKKIGLLVEDMHDVTVDGGGAKLVHHGLQTAFASIRSTDVTFQNFSFD
BfGH110B FPAGRYNFHEAGSTVREYYISNHDQD.....NPKKVGIALEDMKNLTIDGQGSEFVFYGRMIPVSLLRSENCVLKNFSID
BfGlaA LPEGRYDIWPEGAIRKEYYISNTSTEQECPSKVKTVGLMLHEIDDLTIEGNGATLMYHGKMTTIALEHCNGVRINNLHID
BfGH110A LPEGRYDIWPEGAIRKEYYISNTSTEQECPSKVKTVGLMLHEIDDLTIEGNGATLMYHGKMTTIALEHCNGVRINNLHID
BtGH110B FPKGRYDFWAQHAIEKDYYETNTYDV.....NPKILAVLLEQINDLTIDGNGSEFIMHGRMQPFTLDHCRNITLKNFSVD
PdGH110B FSRAFHSEALIA....GAGKGYLDLKFTDQFPYKINEAGILKFQSQLFQASGIKNKDRLKRKQISQDEYKYEYKRVLEFN
BtGH110A YKHPTQTEVEVL....EEGNDYLIVQVHPTSQYRI.VDAQLEWYGDGW.........S...FKNGI....AQSYDRISEMT
BbAgaBb FQVPTVIDLTVEKV..DAGAKTATVYVPEEYNYRL.SGSNIEWYSDSSPYTGATYWT...ASNALP..YVQLYDTKTGLT
SaGH110A YAAPEVIDATVATTGVTDGHAYRVLKIPAGSPYRV.NGTHITWLGETSPATGQPYWS...GVDGLQ..YTQIHDPEAQRT
BfGH110B FEQPHIAQVQVVEN..DPEKG.ITFEPAPWVDYRISKDSVFEGLGEGWVMR...YSW...GIAFDGKTKHVVYNTSD.IG
BfGlaA FERPAGSEIQYRKV..TGGETEVTLH..RDTRYEI.VNGKIRLYGEGWRSN...RNH...CIEYDPDTESFTY....SQG
BfGH110A FERPAGSEIQYRKV..TGGETEVTLH..RDTRYEI.VNGKIRLYGEGWRSN...RNH...CIEYDPDTESFTY....SQG
BtGH110B WEIPLTAQGIVTQS..TSEYLEIEIDS.HQYPYII.ENKRLTFVGEGWKSS...LWA...IMQFDPDTHLVLPNTGDNLG
PdGH110B FALREPEYMAQDIFTGNALRAEKLNGGDVVRIFHPNL.....KAKVGNILVFQAKHRDYPGVVISDSNNVELHNITIHHA
BtGH110A WR..SWSPM.ENLLR.....TVE.LRPN...VLYLQY.KEKPQVGLHTIFQMRDSFRDEVSGFVNRSKGILLENINFYYL
BbAgaBb VR..GDVWT.NPIFQ.NVTGITD.AGNHRLVFSYSSM.SDKLANATGISYQMRQTTRDHPGVFLWKDKDVTLKGIDFRFL
SaGH110A WR..GD....NPLFN.DVAAVTD.LGGRRIRIDYTTA.AR..PADAGLVYQMRLIERTEPGAFIWESKNVTMRSMNAYYL
BfGH110B CP..T............KGAFE.VAPR..RICSPKWKDA..RLVPGTVVAMRGWGRPTPGIFMSHDVNTSLLDVKVHYA
BfGlaA WN..TLS..........ASDARE.IAPGIVRFNTPA....EFMPKAGNTLTVRDIIRDQVGLFILESKNITLSRLQMHYM
BfGH110A WN..TLS..........ASDARE.IAPGIVRFNTPA....EFMPKAGNTLTVRDIIRDQVGLFILESKNITLSRLQMHYM
BtGH110B WR..S.............YDATE.INPGLIRLSDPKKEADKFFPAPGTVLVLRHSTRDHAGIFIYHSMDTKLENVKLFHT
PdGH110B GGMGVIAQRSHNITIKDSK...VSPSKGRIVSTTADATHFVNCTGKIKLIDNLFESQKDDATNIHGVYAAIDKIIDDKTV
BtGH110A GNFGVVCQYSENITVDRCN.FAPRPGSGRTNAGFADFIQVSGCRGMIDIKNSRFIGAHDDPINIHGTHLRVIEFLSDNRL
BbAgaBb HGFGVVGQSTDTITMDGLH.FGTGEGTGRSTAGYADFVQMSGCKGVITVANSSFNPHDDPINVHGTFLQVVEKISDTKI
SaGH110A QSFGVVGQFSENISIDKVN.FAPDPRSGRSTASFADFVQMSGVKGKVSITRSLFDGPHDDPINIHGTYLEVVGKPGPSTL
BfGH110B EGMGLLAQLCEDITLDGFGVCLKGDNDPRYFTTQADATHFSGCKGKIVSKNGLYEGMMDDAINVHGTYLKVIKRVDDHTL
BfGlaA HGLGIVSQYTENITMDRVK.CAPRPDSGRLLAASADMMHFSGCKGKVIIDSCYFAGAQDDPVNVHGTNLRALEKIDAQTL
BfGH110A HGLGIVSQYTENITMDRVK.CAPRPDSGRLLAASADMMHFSGCKGKVIIDSCYFAGAQDDPVNVHGTNLRALEKIDAQTL
BtGH110B CGLGILSQYSKNISFNDVH.IIPNTSKKRVLSGHDDGFHFMGCSGLLKIENCSWAGLMDDPINIHGTCSRIMEVLSPTRI
PdGH110B EIKLQHPQQFGFDFIAPEDELELVHGASLITYETNKVVTSTRVS..............NEVTRVQFIKPFDSRI..KEGD
BtGH110A KLRFMHDQTFGFEAFFKGDDIELVDSRSLLVVGKCKVKEAKLVTP..............REMELTLSSPLSSEVMQQKDL
BbAgaBb KVRYMHNETAGFPSFFVGDQVEFMTKGDMLPVSDSVRVTAVDGPDGQGGDMGAGSGSLTDIVLTLDSAIPSAV.AVNSH
SaGH110A TLAYKHPQTAGFPQFAPGDEVEFATKRTMTPLADAHAQVTAVDGPSGMDH.....TKPLTTMTVTFDRPVPAGV.ETGGT
BfGH110B IGRYMHDQSWGFEWGRPGDDVQFVRSETMELIGKQNQITAIR..PYDKGE.....IRGAREFSITFKEAIDPAINEKSGF
BfGlaA KLRFMHGQSYGFNAYFKGDTVAFVRAATMERFASATVR.........DVR.....RISDRIVEVRFDRDI.PTSLELNHD
BfGH110A KLRFMHGQSYGFNAYFKGDTVAFVRAATMERFASATVR.........DVR.....RISDRIVEVRFDRDI.PTSLELNHD
BtGH110B KCKFMQDMSEGMEWGRPGDETIGFIEHKTMRTVATGKMN.........KFE.....ALNKAEFIIELSVPL.PAGVEA.GY
PdGH110B SVSKVRSYAEVIIKGNIIRKNRARGMLLNSRGKTLIENNYFHTPG.SAILFEGDANFWFEQGGVSDVTIKNNVFENSFYS
BtGH110A VIENVTWTPEVRITNNYFARVPTRGILITTRRKSLIEGNTFYGMQMSGIFVADDGLSWYESGPVHDLTIRQNTFLNCG..
BbAgaBb VVENITYTPEVNIHDNVFKETPTRGILVTTRKKVTIENNLFDGMGMAGIYISNDAQSWYESGPTRDVTIRGNTFRRSG..
SaGH110A VVENITATPSVVISGNVFRNVPTRGILVTTRKPVLITGNRFDGMSMASIYVSADAYQWYESGPVADLTIRGNSFTRPS..
BfGH110B GIENLTWTPEVLFAGNTIRNNRARGTLFSTPKKTVVEDNLFDHTSGTAILLCGDCNGWFETGACRDVTIRRNRFINAL.T
BfGlaA CVENMTCTPEVEIRNCYFTRTSTRGTLVTTPRKVVIENNTYYKTGMSAILIEADAEGWYESGPVKDVLIKGNTFIDCAYN
BfGH110A CVENMTCTPEVEIRNCYFTRTSTRGTLVTTPRKVVIENNTYYKTGMSAILIEADAEGWYESGPVKDVLIKGNTFIDCAYN
BtGH110B VIENLTCTPDAEIRNCHFGSCRARGLLVSTPGKVIIENNVFESSG.SAILIAGDANAWYESGAVKDVLIRNNDFRYPCNS
PdGH110B ...QWGKGIIAVDAGIDDKFKETSRYN.....KNIVIKGNTFKVFDKA.PILNLFSVSNLVFENNIIEKTTEYPERK...
BtGH110A ......EPIISIDPENRE.Y..KGAVH.....KNITIEENYFYMRKNSSCAIRAKAVDGLMIRHNLIYSLDTEKNKE...
BbAgaBb ......SDAILVEPTNPT.VSTTDTVH.....KNMTIEGNTFYVNGNR..VLNAKSVSDLTFRDNKIYRENPDDTVTLGG
SaGH110A ......GPVIFVEPTNQV.IDPATPVH.....HNISVEHNSFDIGDVT..VVNAKSVGGFAFTGNTVRRLDGADHPP...
BfGH110B NMFQFTNAVISIYPEIPN.LKDQQKYFHGGKDGGIVIEDNEFDTFDAP..ILYAKSVDGLIFRNNVIKTNTEFKPFH...
BfGlaA G..GPGHAVIAIHPSNKI.IDAERPVH.....QNIRIEDNTFRTFDYP..VLYAKSTAGLLFRNNTIVRTETFPAVS...
BfGH110A G..GPGHAVIAIHPSNKI.IDAERPVH.....QNIRIEDNTFRTFDYP..VLYAKSTAGLLFRNNTIVRTETFPAVS...
BtGH110B SIYQFCEAVISIDPEIPT.PEQKYPYH.....RNIRIMDNTFHLFDYP..ILFARSVNGLTFSSNTLIRDTTYQPYH...
PdGH110B .........................KYNSLFVINNSDNITISINNILQGFSEGKSQL........LSPTTTYKRAKN.....
BtGH110A .........................SDFIQMYNCNEVTIKENRVQLHHLFK.............................
BbAgaBb DADVALAVGDTRRIDATASVSQVSGSRLFRLNGCKQVVFGGNTYDVGVKAG.IDLANMGASEVNVSDDSAKVGADGLVPV
SaGH110A .........................YTSPLFVFHGSSGIRIARNHYDKGLNTS.VVTD.......................
BfGH110B .........................WNKDRFLLERVTNVKISE...
BfGlaA .........................GNPYVFYLNGCKKAVIEGTVFEGETPRQSIKTENMKRKDLKTTIK............
BfGH110A .........................GNPYVFYLNGCKKAVIEGTVFEGETPRQSIKTENMKRKDLKTTIK............
BtGH110B .........................YRKEGITLEACKSVVISNNKIEGDVLGRIVTIEKMKPSDVKISKNPFFKLKK.....

Figure S5 (preceding page). Amino acid sequence alignment of PdGH110B with the catalytic domains of characterized GH110 enzymes from *Bacteroides fragilis* (Bf), *Bacteroides thetaiotaomicron* (Bt), *Streptomyces avermitilis* (Sa), and *Bifodobacterium bifidum* (Bb). Magenta arrows indicate catalytic residues with their roles indicate by A (acid) or B (base). Purple arrows indicate residues making interactions in the -1 subsite of PdGH110B. Yellow arrows indicate residues making interactions in the +1 subsite, and possibly +1' subsite, of PdGH110B. Grey arrows indicate additional residues that may contribute to a +1' subsite in PdGH110B and/or in other family members. The green box indicates the region in PdGH110B that makes an α-helical finger which extends from one monomer over the active site of the second monomer in the dimer (see Fig. 2C). The secondary structure of Pd110B is indicated above. The numbering corresponds to that of PdGH110B.
